# Oral health status among transgender young adults: a cross-sectional study

**DOI:** 10.1186/s12903-021-01945-x

**Published:** 2021-11-12

**Authors:** Kaur Manpreet, Mohammed B. Ajmal, Syed Ahmed Raheel, Mohammed C. Saleem, Khan Mubeen, Kamis Gaballah, Asmaa Faden, Omar Kujan

**Affiliations:** 1 Kodagu District Hospital, Madikeri, Karnataka India; 2 KGF College of Dental Sciences, Kolar, Karnataka India; 3 Department of Oral Medicine and Radiology, KGF College of Dental Sciences, Kolar, Karnataka India; 4 Government Dental College, Bangalore, India; 5 Department of Oral and Craniofacial Health Sciences, College of Dental Medicine, University of Sharjah, Sharjah, United Arab Emirates; 6 Department of Oral Medicine, College of Dentistry, King Saud University, Riyadh, Saudi Arabia; 7 Oral Diagnostic and Surgical Sciences Division, UWA Dental School, The University of Western Australia, 17 Monash Avenue, Nedlands, WA 6009 Australia

**Keywords:** Transgender, Oral mucosal disorders, Oral findings, Candida, Oral health

## Abstract

**Background:**

Transgender and gender nonconforming (TGNC) people are a marginalized set of the population that continues to experience health care inequalities. This study aimed to assess oral health parameters including *Candida* growth and intensity among TGNC adults.

**Methods:**

This cross-sectional study recruited two subgroups: 40 transgender and 40 control adults. Consented participants were interviewed and clinically examined. Data using the WHO oral health assessment forms were obtained. Samples for *Candida* growth and intensity analysis were collected from the dorsum surface of the tongue.

**Results:**

27.5% of the transgender group was HIV seropositive. Oral nicotine stomatitis and leukoplakia are reported to be the most prevalent intra-oral lesions showing a prevalence of 27.5% and 20%, respectively. The dental and periodontal health parameters of the transgender group were worse than those of the control group. The intensity of *Candida* colonies was significantly higher in the test group (p = 0.014).

**Conclusion:**

Poor oral health and significant oral mucosal disorders were reported in transgender adults that have shown a higher rate of behavioral risk factors such as tobacco and alcohol consumption. Further longitudinal studies in different world regions are warranted to understand the barriers to good oral health in transgender adults and how to implement effective prevention and management strategies.

## Introduction

Transgender and gender nonconforming (TGNC) is an extremely broad concept that means having a gender identity that does not suit the birth-assigned sex of one. In the medical literature, this distinction is also intended to explain natal sex and gender identity with the terms male-to-female (MTF) or female-to-male (FTM). MTF and FTM are used interchangeably with a transgender woman and transgender man, respectively [1, 2]. TGNCs suffer from a gender identity condition medically known as gender dysphoria that is clinically severe distress or disability in social, occupational, or other areas of operation that arises when the gender to which an individual identifies does not suit the sex that has been assigned [2, 3].

The TGNC people face increased social stigma, increased threats of violence, and increased socioeconomic problems including unemployment. Such factors increase their risk of mental health problems such as depression, anxiety, posttraumatic stress disorder, drug use disorder, and attempted suicide [4, 5]. More than 40% of TGNC people in the United States have attempted suicide, compared with fewer than 2% of the general population [6]. People with TGNSs face major obstacles in accessing adequate general health care and specialist gender-affirming services. Such barriers can be categorized into four major categories: hesitation to report, barriers to the health care system, lack of expertise and support from providers, and financial constraints [7, 8]. Overcoming health care barriers including dental treatment for TGNCs, is crucial to reducing health disparities for this population. Lessening the stigma, changing the healthcare system to become more trans-ready, alleviating financial barriers, and increasing the awareness and training of clinicians are all crucial ways in which healthcare is treated [9].

Oral health among TGNCs is complicated by all these factors. This group is likely to display a broad range of oral mucosal lesions with oral candidosis being the most common issue, which has also been identified as conditions preceding manifest syndrome as a sign of various diseases such as HIV infection [10–12]. However, the spectra of the oral mucosal lesions, oral health status, and Candida that are present in this special group of subjects have not been fully reported. This information will help to direct the resources from the health care system and oral health care providers to efficiently deliver the best standard of care to these subjects. Hence, this study aims to report the oral health status and the prevalence of oral mucosal lesions, and isolate Candida species from the oral cavity of transgenders.

## Materials and methods

This is a cross-sectional observational study carried out in the Department of Oral Medicine and Radiology, Government Dental College and Research Institute, Bangalore, India during the period from July 2018 to August 2019, which was reported by the Strengthening the Reporting of Observational Studies in Epidemiology (STROBE) statement [13]. The Research Ethics Committee of the institute approved the study protocol (IRB 00215). All participants provided informed consent, and the study was conducted according to the Declaration of Helsinki.

The study has two groups; Group (A) comprised 40 TGNCs recruited from the non-governmental organization “PAYANA” (Benson Town, Bengaluru, Karnataka, India) for the TGNCs in India. The study included individuals who were self-identified and presently not on any hormonal therapy. The control group (B) of the population comprised 20 males and an equal number of female patients reporting to the Department of Oral Medicine and Radiology, who were of the age group of group (A) study population, not immunocompromised and females who were not pregnant or taking any medications specifically hormonal therapy.

Each individual was then interviewed personally using the World Health Organization (WHO) Oral Health Questionnaire for Adults (2013) [14], and then subjected to a clinical examination that was carried out by MK. The examiner is an experienced dentist and underwent a period of calibration over five clinical sessions to ensure high quality reporting of oral health data. Based on the National Health Interview Survey (NHIS), a history of cigarette smoking and smokeless tobacco was reported. The participant was asked to record their last month's habit experience, whether they only practiced it every day, for a few days, or not at all in the last 30 days. Standard clinical examination was performed using a mouth mirror and Community Periodontal Index (CPI) probe. It has focused on the detection of oral mucosal lesions and the evaluation of the Decayed, Missing, and Filled Teeth Index (DMFT) index, and CPI [15].

Salivary candidal carriage was assessed in a patient with a disease-free tongue. The sample was obtained after oral rinsing with distilled water and drying the area using sterile gauze to eliminate all debris. The dorsal surface of the tongue was gently rubbed with a tube sterile cotton swab to avoid contamination from the rest of the oral cavity. The samples were collected and sent to the Microbiology Department for identification and isolation of Candida species. Swabs were inoculated across the surface of Sabourand’s dextrose agar (SDA) media and incubated at 37 °C for 24–48 h. If present, Candida develops as cream, smooth, pasty convex colonies on SDA. The scoring of the intensity of Candida growth is reported using the method of Gacon et al., [16], as follows: score (0) a lack of fungal growth up to 10 colonies; score (1) scarce growth—10–20 colonies; score (2) intermediate growth—21–50 colonies; score (3) intensive growth—51–100 colonies; and score (4) abundant (confluent) growth—over 100 colonies.

Statistical analysis was performed using SPSS Statistics, version 23.0 (IBM, NY, USA). Mann–Whitney *U* test was used to study the association between variables. Any association with a value p < 0.05 was considered statistically significant.

## Results

The total number of subjects included in this report was 80. The age ranged from 21 to 59 years, with a mean age of the participants of 30.4 years. The general characteristics of the study sample are summarized in Table 1. Only 7.5% (n = 3) of the TGNC group attained a university degree compared to 42.5% (n = 17) of the control group. The social history of the TGNCs revealed that half of them (n = 20) lived on sex work and begging, 45% (n = 18) were homeless, and only 5% (n = 2) were employed as professionals. HIV testing showed that 27.5% (n = 11) of the transgender people were positive. The tobacco consumption history showed that cigarette smoking was not common among the groups.

**Table 1:** General characteristics of the study participants

Variables		Transgenders group	Control group
Age (years) (mean ± SD)		29.7 ± 8.8	31.1 ± 3.5
Education (**P = 0.038**)	Novice (N, %)	4 (10%)	1 (2.5%)
	Primary (N, %)	16 (40%)	9 (22.5%)
	Secondary (N, %)	17 (42.5%)	13 (32.5%)
	University (N, %)	3 (7.5%)	17 (42.5%)
Occupation (**P = 0.0001**)	Homeless (begging) (N, %)	18 (45%)	0 (0%)
	Sex worker (SW) (N, %)	14 (35%)	0 (0%)
	Begging and SW (N, %)	6 (15%)	0 (0%)
	Professionals (N, %)	2 (5%)	25 (62.5%)
	Housewife (N, %)	0 (0%)	15 (37.5%)
HIV seropositive (**P = 0.009**)	Yes (N, %)	11 (27.5%)	0 (0%)
	No (n, %)	29 (72.5%)	40, 100%
Cigarette smoking (P = 0.216)	No	22 (55%)	28 (70%)
	Some days	17 (42.5%)	11 (27.5%)
	everyday	1 (2.5%)	1 (2.5%)
Smokeless tobacco (P = 0.437)	No	28 (70%)	27 (67.5%)
	Some days	8 (20%)	13 (32.5%)
	everyday	4 (10%)	0 (0.0%)
Alcohol (**P = 0.009**)	No	10 (25%)	24 (60%)
	1–4 units	12 (30%)	5 (12.5%)
	> 4 units	18 (45%)	11 (27.5%)
Oral health perception (P = 0.397)	Poor	4 (10%)	0 (0.0%)
	Average	17 (42.5%)	8 (20%)
	Good	16 (40%)	24 (60%)
	Very good	1 (2.5%)	6 (15%)
	Excellent	2 (5%)	2 (5%)
Candida growth (**P = 0.016**)	No	9 (22.5%)	15 (37.5%)
	Scarce	11 (27.5%)	22 (55%)
	Intermediate	10 (25%)	3 (7.5%)
	Confluent	3 (7.5%)	0 (0.0%)
	Intensive	7 (17.5%)	0 (0.0%)

On the other hand, 30% (n = 12) and 32.5% (n = 13) of the TGNCs and control participants, respectively, practiced smokeless tobacco. Alcohol consumption of variable quantity and frequency was reported by three-quarters (n = 30) of the test group. The oral health perception responses among both groups were comparable (p = 0.397). However, oral examination in the present study reported a significantly higher prevalence of oral mucosal lesions (p = 0.026), such as oral ulcers, leukoplakia, nicotine stomatitis, and malignant tumors among the TGNCs compared to the control group (Table 2). During the study period, one transgender person was diagnosed with oral cancer involving the right buccal sulcus area. When evaluating the DMFT index score, a significant difference with a p-value of 0.001 was observed with the TGNCs having a high mean DMFT index score of 8.4 ± 0.91 while the control group had a mean DMFT score of 3.6 ± 1.5. The community periodontal index score did not reveal a statistically significant result (Table 3). The quantitative assessment of the intensity of *Candida* colonies showed statistically significant results with a p-value of 0.016 (Table 1).

**Table 2:** The distribution of oral mucosal lesions among the study participants

	Transgender group	Control group
	Number (%)	Number (%)
No lesions (P = 0.014)	14 (35%)	27 (67.5%)
Oral ulcers (P = 0.637)	6 (15%)	5 (12.5%)
Leukoplakia (P = 0.008)	8 (20%)	0 (0%)
Nicotinic stomatitis (P = 0.764)	11 (27.5%)	8 (20%)
Malignancy (P = 0.017)	1 (2.5%)	0 (0%)

**Table 3:** Dental and periodontal indices for Transgender and control groups

		Transgender group	Control group
		Number (%)	Number (%)
Community periodontal index (P = 0.173)	0	7 (17.5%)	9 (22.5%)
	1	11 (27.5%)	14 (35%)
	2	11 (27.5%)	12 (30%)
	3	7 (17.5%)	4 (10%)
	4	4 (10%)	1 (2.5%)

	Parameter	Mean ± SD	Mean ± SD
DMFT (**P = 0.000**)	D	4.6 ± 1.87	2.42 ± 1.63
	M	2.74 ± 2.3	0.3 ± 0.11
	F	0.85 ± 1.36	1.55 ± 1.54
	DMF	8.4 ± 0.91	3.6 ± 1.5

*D* decay, *M* missing, *F* filled, *SD* standard deviation

Community Periodontal Index: Code 0: health periodontal conditions; Code 1: gingival bleeding on probing; Code 2: calculus and bleeding; Code 3: periodontal pocket 4–5 mm; and Code 4: periodontal pocket ≥ 6 mm

## Discussion

In the present study, the majority of the TGNCs were male transformed females. In most reports in the literature, male transsexuals outnumber female transsexuals. The ratio (MTF: FTM) varies from as low as 1:1 to as high as 8:1 [17, 18]. This difference, however, may be more apparent than real. It has been suggested that today, female transsexuals can dress, work, and live as members of the opposite sex with less social disruption than can the male transsexual [19].

Regarding the educational level among the TGNCs, our study’s results were consistent with a previous study undertaken by Hongal et al. They reported that most eunuchs in their study were illiterate [19]. This disparity in education status could be attributed to social exclusion and abuse faced by these oppressed individuals. In addition, lack of education and an unusual working environment are responsible for transgender inability to get a mainstream job, as reflected in our study where 95% of transgender people indulged in begging or sex-work. Furthermore, our study reported that 27.5% of TGNCs were HIV seropositive. This is consistent with other reports. In the TGNC population, the prevalence of HIV is much higher than the national average. A survey, carried out by the National Center for Transgender Equality was 2.64% HIV-positive for TGNC people, compared to 0.65% for the general population [7, 20].

The self-perception of oral health reflects the knowledge and oral health-related attitude of an individual. In this study, the majority of the TGNCs perceived their oral health to be average; however, most of the control group i.e. males and females, considered it to be good. The latter signifies the level of awareness and ignorance of oral health among the test group, which can be attributed to a lack of education and access to health care services. Various other studies from different countries reported similar results [21–23].

A higher percentage of TGNC individuals indulged in alcohol consumption and smoking at a higher frequency than the control group in the present study. The higher reported rates of these harmful habits could be attributed to occupational and psychosocial stress due to an unfavorable social position, the lack of awareness of the detrimental effect of these habits, scarce material resources, and poor material conditions [22–26]. Our findings are consistent with several studies that reported increased consumption of alcohol and both smoked and smokeless tobacco consumption among TGNC individuals [22–26].

While investigating the oral health status of the study population, DMFT index, community periodontal index, and survey of oral mucosal disorders, our study showed a significantly higher mean DMFT score among TGNCs than the control group DMFT (p = 0.0001). Arjun and coworkers reported poor periodontal health among this specific group of the population [27]. Additionally, oral mucosal disorders such as nicotine stomatitis and oral leukoplakia were observed at a higher frequency among TGNCs, which may correspond to their increased indulgence in behavioral high-risk habits and lack of awareness. Another study reported a higher prevalence of oral submucous fibrosis and leukoplakia among TGNCs [24]. It is already known that transgender subjects suffer from health disparities including access to oral care [7, 8]. This might provide an explanation for the reported differences in the oral health measures between the studied groups. It also highlights the importance of implementing a nationwide strategy to improve access to oral health care for this vulnerable group.

In harmony with previous studies [28–30], we documented a significantly higher score in the quantitative assessment of the intensity of Candida colonies among transgender than the control group. Several reports documented the significant association between both an increased candidal salivary carriage and the presence of clinically detectable candidosis in a patient with reduced CD4 count [29–32].

The increased Candida colonization of TGNC oral cavity can be attributed to various factors, such as increased indulgence in harmful habits, such as smoking, hormone replacement therapy, and increased prevalence of HIV. Cigarette smoke has been suggested to promote C. albicans pathogenesis by promoting the transition from blastospore to hyphal form and increasing the adhesion of this yeast to and proliferation on gingival fibroblasts [33, 34]. Others found increased Candida adherence to buccal epithelial cells in vitro during the various menstrual cycle phases implicating hormonal influence and hormonal factors in candidosis etiology [35]. The increased Candida carriage in patients with HIV infection is associated with changes in immunological reactivity [35].

The present study findings should be interpreted in the right context due to some inherited limitations. The study was carried out at a specific point in time, and a longitudinal follow-up study would be valuable to study the association of increased risk factors, such as tobacco and alcohol consumption and the prevalence of oral mucosal disorders. The study has a relatively small sample size. We used a convenient sample given the difficulty in recruiting TGNCs to participate in the study. Additionally, the control group did not match for the age and gender of the participants; however, this study was not designed to be a case–control study. We also acknowledge that our study might not represent all Indian TGNCs since our data is obtained from a single geographic region and the study did not exhaustively examine the sociological background of our participating TGNSs. However, our observation of the poor sociological characteristics of TGNCs is similar to that reported in other parts of India and Pakistan [36]. In India, despite the legal recognition of this group, social discrimination and stigma have created a barrier to schooling or accessing secondary or tertiary education, eventually leading to poor health literacy [36].

## Conclusion

Our study findings highlighted the presence of poor oral health and significant oral mucosal disorders in transgender adults. These were associated with a higher rate of harmful social habits. Additionally, Candida species colonization of the oral cavity was significantly higher among the transgenders. Our study underlines the importance of paying attention to this vulnerable group by oral health providers and the health system to offer the best standard of care. Nonetheless, further longitudinal studies in different world regions are warranted to understand the barriers to good oral health in the transgenders and how to implement effective prevention and management strategies for a better quality of life.

## Data Availability

Further information on the data set and materials is available from the corresponding author upon reasonable request.

## Abbreviations

CPI: Community Periodontal Index
DMFT: Decayed, missing, and filled teeth
FTM: Female-to-male
HIV: Human immunodeficiency virus
MTF: Male-to-female
TGNC: Transgender and gender nonconforming
WHO: World Health Organization
